# *Rasnitsynala sigambrorum* gen. et sp. n., a small odonatopterid (“Eomeganisoptera”, “Erasipteridae”) from the early Late Carboniferous of Hagen-Vorhalle (Germany)

**DOI:** 10.3897/zookeys.130.1458

**Published:** 2011-09-24

**Authors:** Wolfgang Zessin, Carsten Brauckmann, Elke Gröning

**Affiliations:** 1 Lange Straße 9, 19230 Jasnitz, Germany; 2 Clausthal University of Technology, Institute of Geology and Paleontology, Leibnizstraße 10, 38678 Clausthal-Zellerfeld, Germany

**Keywords:** Odonatoptera, “Eomeganisoptera”, “Erasipteridae”, gen. et sp. n., early Late Carboniferous, Namurian B, Marsdenian, Hagen-Vorhalle, Germany

## Abstract

Besides *Erasipteroides valentini* (Brauckmann in Brauckmann, Koch & Kemper, 1985), *Zessinella siope* Brauckmann, 1988, and *Namurotypus sippeli* Brauckmann & Zessin, 1989, *Rasnitsynala sigambrorum* **gen. et sp. n.** is the fourth species of the Odonatoptera from the early Late Carboniferous (Early Pennsylvanian: Namurian B, Marsdenian) deposits of the important Hagen-Vorhalle Konservat-Lagerstätte in Germany. With its wing-span of about 55 mm it is unusually small even for the “Eomeganisoptera”. Its venation resembles other small “Eomeganisoptera”, in particular *Zessinella siope*. This is why it is here assigned to the probably paraphyletic “Erasipteridae” Carpenter, 1939.

## Introduction

Up to now three species of Odonatoptera: Neodonatoptera have been described from early Late Carboniferous (Early Pennsylvanian: Namurian B, Marsdenian) deposits of the important Konservat-Lagerstätte of Hagen-Vorhalle in Germany, i.e. *Erasipteroides valentini* (Brauckmann in Brauckmann, Koch & Kemper, 1985), *Zessinella siope* Brauckmann, 1988 (both “Eomeganisoptera”: “Erasipteridae”) and *Namurotypus sippeli* Brauckmann & Zessin, 1989 (Euodonatoptera: Meganisoptera). Due to the nearly completely preserved specimens, in particular *Erasipteroides valentini* and *Namurotypus sippeli* enlarged the knowledge of the morphology and presumed behaviour of Late Palaeozoic Odonatoptera essentially (Brauckmann and Zessin 1989, Bechly et al. 2001, Zessin 2008b). With a wing-span of about 17 cm and 32 cm, respectively, these two species are both large when compared with *Zessinella siope*, which only reaches a wing-span of about 8 cm. *Rasnitsynala sigambrorum* gen. et sp. n. is the fourth species of the Odonatoptera of this collecting site. With its wing-span of only 55 mm it is unusually small even for the “Eomeganisoptera”.

The previously described species from Hagen-Vorhalle are among several findings of Odonatoptera from other Late Carboniferous (Pennsylvanian) collecting sites, in particular in Germany (Zessin 1983, Brauckmann 1983) and Argentina (Riek and Kukalová-Peck 1984) which were discovered in the years between 1980 and 1990. After a long period of stagnation of scientific investigations in Odonatoptera, these new materials gave rise to further interest in this group. Subsequently several contributions have been published, in particular during the last decade, including descriptions of newly discovered materials of already described taxa (Beckemeyer 2000, 2006, Beckemeyer and Hall 2007) as well as of new species and genera (Zessin 2006, Zhang et al. 2006, Ren et al. 2008, Nel et al. 2008, Prokop and Nel 2008, Zessin and Brauckmann 2010), detailed morphological interpretations (Kukalová-Peck 2009), phylogenetic studies (Bechly et al. 2001, Jarzembowski and Nel 2002, Kukalová-Peck 2009), revisions of larger parts and compilations (Zessin 2008a, Nel et al. 2009), and even research concerning the presumed flight adaptations and behaviour (Wootton et al. 1998, Wootton and Kukalová-Peck 2000, Bechly et al. 2001, Zessin 2008b).

## Material, methods and terminology

The nomenclature of the wing venation follows Redtenbacher (1886), Riek and Kukalová-Peck (1984), and Bechly (1996). Abbreviations used in this contribution are: PC, Precosta; CA+ = Costa anterior; CP– = Costa posterior; ScA+ = Subcosta anterior; ScP– = Subcosta posterior; R = Radius; RA+ = Radius anterior; RP– = Radius posterior; M = Media; MA+ Media anterior; MP– = Media posterior; Cu = Cubitus; CuA+ = Cubitus anterior; CuP– = Cubitus posterior; A = Analis; AA+ = Analis anterior; AP– = Analis posterior; JuA+ = Jugalis anterior. Attached + and – indicate the corrugation of the wing. The corrugation is not distinctly preserved in *Rasnitsynala sigambrorum* gen. et sp. n. The nomenclature of the areas between the main veins follows Zessin (1987).

The photograph and drawing of the holotype was prepared by digital camera Nikon D3 and Corel Draw 12.

## Systematic palaeontology

**Remarks.** The systematics of early Odonatoptera as used in the present article follows Bechly (1996), Bechly et al. (2001) and Zessin (2008a). Rohdendorf and Rasnitsyn (1980) as well as Rasnitsyn and Pritykina (2002) introduced a different nomenclature based upon the name of a type genus of each group, a nomenclatural method that has long been used in botany. Since these two points of view use different ranks they cannot easily be compared at each level, at least in early Odonatoptera.

**Odonatoptera Martynov, 1932 = Libellulidea sensu Rasnitsyn & Pritykina, 2002**

**Neodonatoptera Bechly, 1996**

**“Eomeganisoptera” Rohdendorf, 1962**

**“Erasipteridae” Carpenter, 1939**

**Remarks.** As shown by Brauckmann and Zessin (1989) and Bechly et al. (2001), both the “Eomeganisoptera” and “Erasipteridae” are most probably paraphyletic.

### Rasnitsynala gen. n.

urn:lsid:zoobank.org:act:E891FF93-B6B1-4E57-B5DE-0019871FEAE2

http://species-id.net/wiki/Rasnitsynala

#### Type (and only known) species.

*Rasnitsynala sigambrorum* gen. et sp. n.

#### Diagnosis.

As for the type species (due to the temporarily monospecific status). Thus far the physically smallest genus of the “Erasipteridae” with typical morphology of distal venation: kink between CuA+ and CuP– very short, ICu area forming a high triangle, and angle between AA1+ and CuA+ nearly 45°.

#### Discussion.

**Figure 1.**
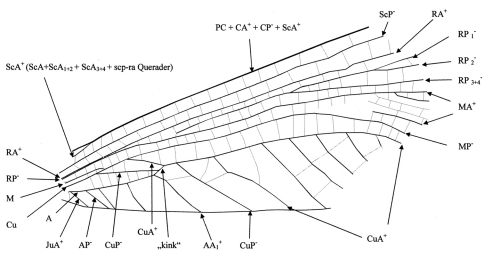
*Aulertupus tembrocki* Zessin & Brauckmann 2010, holotype, left mesothoracic wing, preserved length 63 mm; Late Carboniferous (Pennsylvanian): Westphalian C/D; Morris, Mazon Creek, Illinois, USA, nomenclature of wing venation. From Zessin and Brauckmann (2010).

In its general characters (small size, venation, and cell configuration) *Rasnitsynala* gen. n. closely resembles the previously described genera of the “Erasipteridae” as defined by Brauckmann and Zessin (1989), in particular *Zessinella* Brauckmann, 1988, and is therefore placed in this family. It differs from all other “Erasipteridae” in the basal course of CuA+ and CuP– with very short kink. In the mesothoracic wing, AA1+ runs steeply towards the posterior margin, only similar to *Aulertupus tembrocki* Zessin & Brauckmann, 2010 (Fig. 1). The basal intercubital area (ICu area) resembles a relatively high triangle with its point downwards. In *Erasipteron* Pruvost, 1933, *Erasipteroides* Brauckmann and Zessin, 1989, *Erasipterella* Brauckmann, 1983 and *Zessinella* this area is more a parallelogram with relatively short branch-lines. *Whalleyala* Brauckmann and Zessin, 1989 is much larger, and AA1+ is subparallel to the distal Cu; in contrast AA1+ forms an angle of about 45° with the distal Cu, in particular CuA+, in *Rasnitsynala* gen. n. Additionally *Campyloptera* Brongniart, 1893 again differs distinctly in this distal part of the venation as well as in its larger size.

#### Etymology.

In honour of Professor Dr Alexandr P. Rasnitsyn (Moscow).

### Rasnitsynala sigambrorum sp. n.

urn:lsid:zoobank.org:act:2DA218E6-D010-45C2-9763-5C78DC7F0758

http://species-id.net/wiki/Rasnitsynala_sigambrorum

Figs 2 –3

Zessin 2008a: 5 – kleine, neue unbeschriebene Art von Vorhalle (= small, yet undescribed new species from Vorhalle)

#### Holotype.

Former Sippel collection no. 182 A and B, now collection of the LWL-Museum für Naturkunde, Westfälisches Landesmuseum mit Planetarium in Münster (Germany), positive imprint: no. WMf.N P27781, negative imprint: no. WMf.N N3182B.

#### Type locality.

Abandoned brickyard quarry in Hagen-Vorhalle, North Rhine-Westphalia, Germany (topographic map 1 : 25,000 sheet no. 4610 Hagen/Westfalen; 51°22.88'N; 007°26.77'E, ~115 m a.s.l.).

#### Stratum typicum.

Early Late Carboniferous (Early Pennsylvanian: late Namurian B, late Marsdenian), Ziegelschiefer Formation.

#### Diagnosis.

Thus far the smallest species of the „Erasipteridae“ with the following main characters: Mesothoracic wing: (i) Length: 27 mm; (ii) anterior margin very slightly convex; (iii) ScP– very long, nearly reaching apex; (iv) subcostal area (Sc area) distally very narrow between ScP– and “PC/CA+/CP–/ScA+”; (v) AA1+ short, forming an angle of about 45° with Cu and CuA+; (vi) basal parts of CuA+ and CuP– rather long; (vii) basal intercubital area (ICu area) a relatively high triangle with its point downwards; (viii) kink between CuA+ and CuP– very short. Metathoracic wing: (i) Length: 22 mm; (ii) anterior margin very slightly convex. Body: (i) Preserved length (without head and posterior 3 abdominal segments): 24.5 mm, estimated total length: 32 mm; (ii) abdominal segments narrow and long.

**Figure 2.**
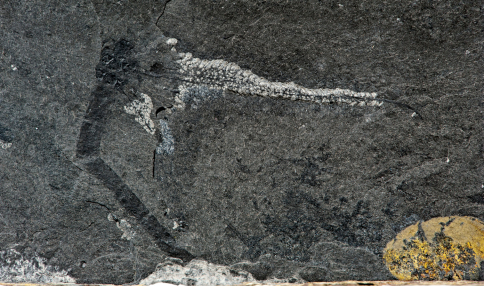
*Rasnitsynala sigambrorum* gen. et sp. n., holotype, former Sippel collection no. 182 A, now collection of the LWL-Museum für Naturkunde, Westfälisches Landesmuseum mit Planetarium in Münster (Germany), positive imprint WMf.N P27781, nearly complete specimen; Early Late Carboniferous (Early Pennsylvanian: Namurian B, Marsdenian); abandoned brickyard quarry, Hagen-Vorhalle (Germany). Preserved length (= without head and posterior three segments): 24,5 mm, length of mesothoracic wing: 27 mm. Photograph by LWL- Museum für Naturkunde, Berenika Oblonczyk.

**Figure 3.**
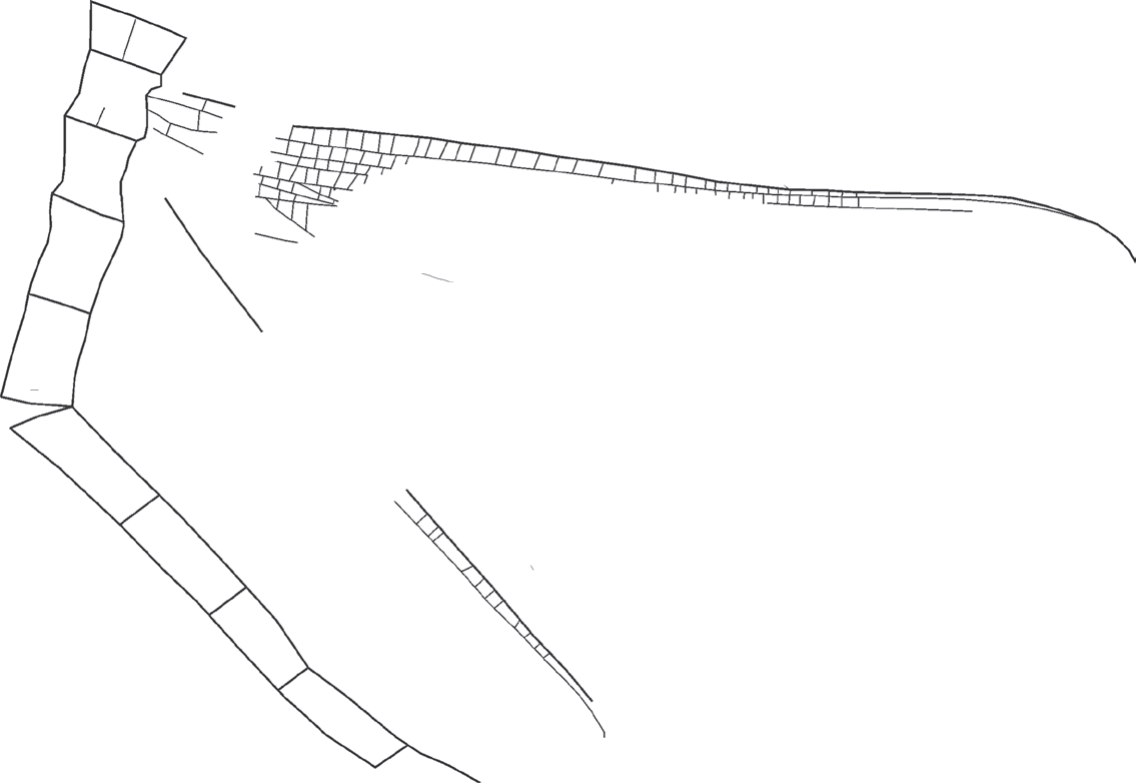
Drawing of *Rasnitsynala sigambrorum* gen. et sp. n., holotype, former Sippel collection no. 182 A, now collection of the LWL-Museum für Naturkunde, Westfälisches Landesmuseum mit Planetarium in Münster (Germany), positive imprint WMf.N P27781, nearly complete specimen; Early Late Carboniferous (Early Pennsylvanian: Namurian B, Marsdenian); abandoned brickyard quarry, Hagen-Vorhalle (Germany). Body structure schematized. Preserved length (= without head and posterior three segments): 24.5 mm, length of mesothoracic wing: 27 mm. Drawing by Wolfgang Zessin, Jasnitz (Germany).

#### Preservation.

The rather well preserved fossil lies in dorsal view. The head with antennae, legs, the posterior three abdominal segments and the left wings are lacking as well as the posterior regions of the right wings. Nevertheless, the preserved parts of the right wings clearly exhibit the main diagnostic features. The original corrugation is nearly completely flattened by tectonic processes but can be reconstructed by comparison with the characters of the ground plan (Kukalová-Peck 2009). The body is compressed, too, and a part of the thoracic segments seems to have been split off. Possibly the right mesothoracic wing was basally disconnected.

#### Measurements (in mm).

(i) Length of mesothoracic wing, 27; (ii) length of metathoracic wing, 22; (iii) preserved length of body (without posterior three abdominal segments), 24.5, estimated total length, 32.

#### Description.

Mesothoracic wing: Anterior margin very slightly convex; ScP– very long, nearly reaching apex; subcostal area (Sc area) distally very narrow between ScP– and complex PC/CA+/CP–/ScA+; AA1+ short, forming an angle of about 45° with Cu and CuA+; basal parts of CuA+ and CuP– rather long; basal intercubital area (ICu area) resembling a relatively high triangle with its point downwards; kink between CuA+ and CuP– very short. Metathoracic wing: Anterior margin very slightly convex, nearly straight. Body: Abdominal segments slender and long.

#### Etymology.

After the Latin name of the Germanic tribe Sigambri (or Sugambri) who inhabited the type region of the species.

#### Discussion.

With a wing-span of about 55mm, *Rasnitsynala sigambrorum* gen. et sp. n. represents the smallest species known to date of the presumably paraphyletic “Erasipteridae”. It shows some characters already known from the very plesiomorphic Geroptera: Eugeropteridae (metathoracic wing of *Eugeropteron lunatum* Riek in Riek & Kukalová-Peck, 1984) or Meganisoptera: Aulertupidae (*Aulertupus tembrocki*), e.g. (i) the morphology of the intercubital area (ICu area) and (ii) the course of AA1+. The abdominal morphology is unknown in most Palaeozoic Odonatoptera and cannot be compered. In *Rasnitsynala sigambrorum* gen. et sp. n. it is extremely slender and longer than the wings. It is even relatively longer and narrower than in the contemporaneous *Namurotypus sippeli* and *Erasipteroides valentini* but seems to be similar in *Zessinella siope*, again of the same age and locality. This character is common in several Recent Zygoptera. It demonstrates that the basic ground plan of Odonatoptera was already established in the very early phase of their evolution, presumably long before the Namurian B (about 319 m.y. ago).

## Conclusion

Though extremely rare, Late Palaeozoic Odonatoptera are famous for the giant size of some of their species with a wing-span of more than 70 cm. On the other hand, *Rasnitsynala sigambrorum* gen. et sp. n. shows once more that they include small relatives, too. Previous collections were largely accidental or concentrated on easily recognizable moderate to large insects. We expect that focussing further prospection onto small and tiny species will significantly broaden our knowledge of early Hexapoda. Another vivid example for this is most recently given by Ilger and Brauckmann (2011).

## Supplementary Material

XML Treatment for Rasnitsynala

XML Treatment for Rasnitsynala sigambrorum
